# Optimizing Age Penalty in Time-Varying Networks with Markovian and Error-Prone Channel State

**DOI:** 10.3390/e23010091

**Published:** 2021-01-10

**Authors:** Yuchao Chen, Haoyue Tang, Jintao Wang, Jian Song

**Affiliations:** 1 Department of Electronic Engineering, Tsinghua University, Beijing 100084, China; cyc20@mails.tsinghua.edu.cn (Y.C.); tanghaoyue13@tsinghua.org.cn (H.T.); jsong@tsinghua.edu.cn (J.S.); 2 Beijing National Research Center for Information Science and Technology (BNRist), Beijing 100084, China; 3 Research Institute of Tsinghua University in Shenzhen, Shenzhen 518057, China

**Keywords:** age of information, cross-layer design, constrained Markov decision process

## Abstract

In this paper, we consider a scenario where the base station (BS) collects time-sensitive data from multiple sensors through time-varying and error-prone channels. We characterize the data freshness at the terminal end through a class of monotone increasing functions related to Age of information (AoI). Our goal is to design an optimal policy to minimize the average age penalty of all sensors in infinite horizon under bandwidth and power constraint. By formulating the scheduling problem into a constrained Markov decision process (CMDP), we reveal the threshold structure for the optimal policy and approximate the optimal decision by solving a truncated linear programming (LP). Finally, a bandwidth-truncated policy is proposed to satisfy both power and bandwidth constraint. Through theoretical analysis and numerical simulations, we prove the proposed policy is asymptotic optimal in the large sensor regime.

## 1. Introduction

The requirements for data freshness in numerous emerging applications are becoming stricter [1,2]. However, the limited resources and bandwidth, together with the fading and error-prone channel characteristics, prevent the control terminal from obtaining the newest information. Moreover, the traditional optimization goals like low delay and high throughput cannot fully characterize the requirement of data freshness. Therefore, it is necessary to introduce new metrics to capture data freshness in such systems and design strategies to optimize the system performance in the presence of resource and environment restrictions.

Recently, a popular metric, Age of information (AoI), has been proposed in [3] to measure the data freshness. Since then, the optimization of age performance under different systems has been a research hotspot. The simple point-to-point system model has been studied in [3,4,5,6,7,8,9,10,11]. When update packets are generated by external sources and are queued in a buffer before transmission, queuing theory can be used to analyze the performance of such system, see, e.g., in, [3,4,5,6,7,8]. In [3], it is shown that the optimum packet generation rate of a first-come-first-served (FCFS) system should achieve a trade-off between throughput and delay. In [8], dynamic pricing is used as an incentive to balance the AoI evolution and the monetary payments to the users. Other studies [9,10,11] consider the generate-at-will system without a queue. Energy constraints are studied in [10,11] to find the trade-off between the age performance and energy consumption. In [11], both offline and online heuristic policies are proposed to optimize the average AoI, which outperform the greedy approach.

Apart from the point-to-point systems, scheduling strategies in the multi-user networks are studied in [12,13,14,15,16,17,18,19]. Different scheduling policies are studied in [12] to minimize the average AoI performance through unreliable channels, and Maatouk et al. verify the asymptotic optimality of Whittle’s index policy by setting an upper bound on the maximum AoI [13]. When each user has a minimum throughput requirement, the authors of [14] propose the Drift-Plus-Penalty policy using Lyapunov analysis to minimize the average AoI performance. In [17], a slotted ALOHA algorithm is proposed to minimize the average AoI, whose performance is verified in the large system regime.

Notice that in many general scenarios, like remote estimation [20,21], the proper evaluation of data freshness is a function of AoI instead of AoI itself. Therefore, the metric of Cost of Update Delay (CoUD) in [7] and age penalty function in [9] have been proposed to measure data freshness in a general setting. However, those works focus on minimizing data freshness in a single-user network, and the multi-user model is rarely considered. To fill this gap, we study a scenario where the base station (BS) collects time-sensitive data from multiple sensors through time-varying channels. We generalize our previous work [22] by considering a more realistic time-varying channel with packet loss and a more general age penalty measurement to model different application scenarios. The main contributions of the paper are listed as follows.

- We study the scheduling strategy for age penalty minimization in multi-sensor bandwidth constrained networks through time-varying and error-prone channel links with power limited sensors. To study a practical network, we model the channel to be a finite-state ergodic Markov chain. The packet loss probability and power consumption depend on the current channel state. Unlike previous work, we model the effect of data staleness in different scenarios via a class of monotone increasing function related to AoI.
- Through relaxing the hard bandwidth constraint and Lagrange multipliers, we decouple the multi-sensor optimization problem into several single-sensor constrained Markov decision process (CMDP) problems. To deal with the potential infinite age penalty, we deduce the threshold structure of the optimal policy and then obtain the approximate optimal single-sensor scheduling decision by solving a truncated linear programming (LP). We prove the solution to the LP is asymptotic optimal when the truncated threshold is sufficiently large.
- The sub-gradient ascend method is applied to find the optimal Lagrange multiplier to satisfy the relaxed bandwidth constraint. Finally, we propose the truncated stationary policy to meet the hard bandwidth constraint. The average performance of the strategy is verified through theoretical analysis and numerical simulations.

The remainder of this paper is organized as follows. The network model, the AoI metric, and the age penalty function are introduced in Section 2. In Section 3, we formulate the primal scheduling problem, and then decouple it into independent single-sensor problems through bandwidth relaxation and Lagrange multipliers. The approximate optimal policy for single-sensor problem is obtained in Section 4 by solving an LP. In Section 5, the asymptotic optimal truncated policy is proposed. Finally, Section 6 provides simulation results to verify the performance of the proposed truncated policy, and Section 7 draws the conclusion.

Notations: All the vectors and matrices are denoted in boldface. The probability of A given B is denoted as Pr(A|B). Let $E_{\pi }\left[X\right]$ be the expectation of variable *X* given π. The cardinality of a set Ω is written as |Ω|.

## 2. System Model

### 2.1. Network Model

In this work, we consider a BS collecting update packets from *N* time-sensitive sensors through time-varying channels. The time is slotted, and t∈{1,2,...,T} is used to denote the current slot index. Define $u_{n}\left(t\right)$ to be the scheduling decision for sensor *n* in slot *t*, where $u_{n}\left(t\right)=1$ means the sensor *n* chooses to send the newest packet, while $u_{n}\left(t\right)=0$ means idling the channel link. Assume all the scheduling behaviors take place at the beginning of each slot and the packet transmission delay through all the channel links is one slot. Due to the limited bandwidth capacity of the BS, the total number of sensors to be scheduled in one slot cannot be larger than *M*. Here, we assume M<N so that the problem is nontrivial:

(1) $$\sum_{n=1}^{N}u_{n}\left(t\right)\leq M,\;\forall t.$$

To model the time varying effect, we assume that the channel link connecting the BS and each sensor *n* is an ergodic *Q*-state Markov chain. Denote $q_{n}\left(t\right)\in \left\{1,2,\cdots ,Q\right\}$ to be the channel state of link *n* in slot *t*. Without loss of generality, we assume that the channel state becomes more noisy as the state index becomes larger. Denote $P_{n}=\left\{p_{ij}^{n}\right\}$ to be the Markov state transition matrix of link *n*, and the entry $p_{ij}^{n}$ means the probability of changing into state *j* in the next slot given the current state *i*, i.e.,

(2) $$p_{ij}^{n}≜\mathrm{Pr}\left\{q_{n}\left(t+1\right)=j|q_{n}\left(t\right)=i\right\}.$$

Due to different channel states, the sensors should use different energy for both saving energy and successful decoding of the packet at the receiver. We denote w(q) to be the energy consumption for scheduling when the channel state is *q*. Notice that the energy consumption tends to be larger as the channel state becomes more noisy to combat the channel fading, i.e., w(1)<w(2)<...<w(Q). Besides, due to the power limit of each sensor *n*, the total average power consumption cannot exceed the upper bound, denoted by $E_{n}$, i.e.,

(3) $$\lim_{T\to \infty }\frac{1}{T}E_{\pi }\left[\sum_{t=1}^{T}u_{n}\left(t\right)w\left(q_{n}\left(t\right)\right)\right]\leq E_{n},\;\forall n,$$

where π is a scheduling policy.

Given channel state *q*, we assume that there exists the probability of packet loss $\varepsilon_{n,q}$ through link *n* due to decoding error or inaccurate estimation.

### 2.2. Age of Information and Age Penalty

In the network described above, the BS wishes to obtain the freshest information for further process or accurate prediction. We model the data staleness at the terminal end as a monotone increasing age penalty function f(·) of Age of information (AoI). By definition, AoI is the difference between the current time slot and the time slot that the freshest data received by the BS is generated by the sensor [3].

Let $x_{n}\left(t\right)$ be the AoI of sensor *n* in slot *t*. According to the definition, if the sensor is scheduled in slot *t* and there is no packet loss, then $x_{n}\left(t+1\right)=1$; otherwise, $x_{n}\left(t+1\right)=x_{n}\left(t\right)+1$. In conclusion, the AoI evolves as follows,

(4) $$\begin{array}{c} \mathrm{Pr}(x_{n}\left(t+1\right)=x^{\prime }\left|x_{n}\left(t\right)=x,u_{n}\left(t\right)=u\right)=\left\{\begin{array}{cc} 1-\varepsilon_{n,q_{n}\left(t\right)}, & x^{\prime }=1,u=1;\\ \varepsilon_{n,q_{n}\left(t\right)}, & x^{\prime }=x+1,u=1;\\ 1, & x^{\prime }=x+1,u=0;\\ 0, & \mathrm{otherwise}.\; \end{array}\right. \end{array}$$

## 3. Problem Formulation and Decomposition

### 3.1. Problem Formulation

For given network, we measure the data freshness at the terminal side by computing the average age penalty under scheduling policy π, denoted by J(π), i.e.,

(5) $$J\left(\pi \right)=\lim_{T\to \infty }\frac{1}{NT}E_{\pi }\left[\sum_{t=1}^{T}\sum_{n=1}^{N}f\left(x_{n}\left(t\right)\right)|x\left(0\right)\right],$$

where $x\left(0\right)=[x_{1}\left(0\right),x_{2}\left(0\right),...,x_{N}\left(0\right)]$ states the initial AoI of the system. In this work, we assume that the system is synchronized with all the sensors at the beginning, i.e., $x_{n}\left(0\right)=1,\;\forall n$, and thus omit x(0) in the further analysis.

We denote $\Pi_{\mathrm{CP}}$ to be the set of all possible causal policies whose decisions are only based on current and historic information while satisfying both bandwidth and power constraints. Then, our goal is to optimize Equation (5) by choosing a scheduling policy $\pi \in \Pi_{\mathrm{CP}}$. Therefore, the primal optimization problem can be written as

**Problem** **1** (Primal Scheduling Problem)**.**

(6a) $$\min_{\pi \in \Pi_{\mathrm{CP}}}\lim_{T\to \infty }\frac{1}{NT}E_{\pi }\left[\sum_{n=1}^{N}\sum_{t=1}^{T}f\left(x_{n}\left(t\right)\right)\right],$$

(6b) $$\begin{array}{cc} s.t.\; & \sum_{n=1}^{N}u_{n}\left(t\right)\leq M,\;\forall t, \end{array}$$

(6c) $$\begin{array}{cc} \; & \lim_{T\to \infty }\frac{1}{T}E_{\pi }\left[\sum_{t=1}^{T}u_{n}\left(t\right)w\left(q_{n}\left(t\right)\right)\right]\leq E_{n},\;\forall n. \end{array}$$

### 3.2. Problem Decomposition

Notice that Equation (6b) is an integer programming where the exponential growth rate of state and action space set obstacles in solving Problem 1. Therefore, we formulate a relaxed version of Problem 1, where the primal bandwidth constraint in every slot is replaced by a time-average bandwidth constraint. We will then show that the relaxed problem can be solved by sensor level decoupling, which greatly reduces the cardinality of the state and action space.

**Problem** **2** (Relaxed Primal Scheduling Problem)**.**

(7a) $$Age_{R}^{*}=\min_{\pi \in \Pi_{\mathrm{CP}}}\lim_{T\to \infty }\frac{1}{NT}E_{\pi }\left[\sum_{n=1}^{N}\sum_{t=1}^{T}f\left(x_{n}\left(t\right)\right)\right],$$

(7b) $$\begin{array}{cc} s.t.\; & \lim_{T\to \infty }\frac{1}{T}E_{\pi }\left[\sum_{t=1}^{T}\sum_{n=1}^{N}u_{n}\left(t\right)\right]\leq M, \end{array}$$

(7c) $$\begin{array}{cc} \; & \lim_{T\to \infty }\frac{1}{T}E_{\pi }\left[\sum_{t=1}^{T}u_{n}\left(t\right)w\left(q_{n}\left(t\right)\right)\right]\leq E_{n},\;\forall n. \end{array}$$

Denote $\pi_{R}^{*}$ to be the optimal policy of Problem 2. The following theorem ensures that the optimal policy of the relaxed problem is composed of several local optimal policies $\pi_{n}^{*}$, each of which depends on its own channel state and AoI evolution regardless of others.

**Theorem** **1.** The optimal policy of Problem 2 can be decoupled into local optimal policies, i.e., $\pi_{R}^{*}=\pi_{1}^{*}⊗\pi_{2}^{*}⊗\cdots ⊗\pi_{N}^{*}$. Each of the local policy $\pi_{n}^{*}$ has the following properties.

$$\begin{array}{cc} \; & Age_{R}^{*}=\lim_{T\to \infty }\frac{1}{NT}\sum_{t=1}^{T}\sum_{n=1}^{N}E_{\pi_{n}^{*}}\left[f\left(x_{n}\left(t\right)\right)\right],\\ \; & \lim_{T\to \infty }\frac{1}{T}\sum_{t=1}^{T}\sum_{n=1}^{N}E_{\pi_{n}^{*}}\left[u_{n}\left(t\right)\right]\leq M,\\ \; & \lim_{T\to \infty }\frac{1}{T}E_{\pi_{n}^{*}}\left[\sum_{t=1}^{T}u_{n}\left(t\right)w\left(q_{n}\left(t\right)\right)\right]\leq E_{n},\;\forall n. \end{array}$$

The proof of Theorem 1 is provided in Appendix A.

In order to find the local optimal polices, we introduce a Lagrange multiplier λ≥0 to eliminate the relaxed bandwidth constraint, and the Lagrange function is as follows,

$$\begin{array}{c} L\left(\pi ,\lambda \right)=\lim_{T\to \infty }\frac{1}{NT}\sum_{n=1}^{N}\sum_{t=1}^{T}E_{\pi_{n}}\left[f\left(x_{n}\left(t\right)\right)+\lambda u_{n}\left(t\right)-\lambda \frac{M}{N}\right], \end{array}$$

where the Lagrange multiplier λ can be seen as a scheduling penalty which will increase the function value if there are more than *M* sensors to be scheduled per slot in average.

For fixed λ, we can now further decouple the relaxed scheduling problem into *N* single-sensor cross-layer designs, each of which has the corresponding power constraint in Equation (7c):

**Problem** **3** (Single-Sensor Decoupled Problem)**.**

(8a) $$\min_{\pi_{n}\in \Pi_{\mathrm{CP}}}\lim_{T\to \infty }\frac{1}{T}E_{\pi_{n}}\left[\sum_{t=1}^{T}\left(f\left(x_{n}\left(t\right)\right)+\lambda u_{n}\left(t\right)\right)\right],$$

(8b) $$\begin{array}{cc} s.t.\;\; & \lim_{T\to \infty }\frac{1}{T}E_{\pi_{n}}\left[\sum_{t=1}^{T}u_{n}\left(t\right)w\left(q_{n}\left(t\right)\right)\right]\leq E_{n}. \end{array}$$

As the resolution of each decoupled problem is independent of *n*, we omit the subscript *n* in further analysis.

## 4. Single-Sensor Problem Resolution

### 4.1. Constrained Markov Decision Process Formulation

First, we notice that the decoupled problem is a constrained Markov decision process of which the elements (S,A,Pr(·|·),c(·)) and constraint are explained as follows.

- State Space: The state of each sensor consists of two parts: the current AoI x(t) and channel state q(t). Thus, S={x×q} is infinite but countable.
- Action Space: There are two possible actions in the action space A for the scheduling policy, denoted by u(t). Action u(t)=1 means the sensor chooses to schedule while u(t)=0 means idling. Notice that here u(t) does not need to satisfy the bandwidth constraint.
- Probability Transition Function: According to Equations (2) and (4), the probability transition function can be written out as follows.
  $$\begin{array}{cc} \; & \mathrm{Pr}\left(\left(x+1,q^{\prime }\right)|\left(x,q\right),u=0\right)=p_{qq^{\prime }},\\ \; & \mathrm{Pr}\left(\left(x+1,q^{\prime }\right)|\left(x,q\right),u=1\right)=p_{qq^{\prime }}\varepsilon_{n,q},\\ \; & \mathrm{Pr}\left(\left(1,q^{\prime }\right)|\left(x,q\right),u=1\right)=p_{qq^{\prime }}\left(1-\varepsilon_{n,q}\right). \end{array}$$
- One-Step Cost: The one-step cost consists of two parts: the age penalty growth and scheduling penalty, which can be computed by
  (9) $$c_{x}\left(x\left(t\right),q\left(t\right),u\left(t\right)\right)=f\left(x\left(t\right)\right)+\lambda u\left(t\right).$$
  And the one-step power consumption is
  (10) $$c_{E}\left(x\left(t\right),q\left(t\right),u\left(t\right)\right)=u\left(t\right)w\left(q\left(t\right)\right).$$

Now our goal is to optimize the following average one-step cost,

(11) $$\min_{\pi \in \Pi_{\mathrm{CP}}}\lim_{T\to \infty }\frac{1}{T}E_{\pi }\left[\sum_{t=1}^{T}c_{x}\left(x\left(t\right),q\left(t\right),u\left(t\right)\right)\right],$$

under the following average power constraint,

(12) $$\lim_{T\to \infty }\frac{1}{T}E_{\pi }\left[\sum_{t=1}^{T}c_{E}\left(x\left(t\right),q\left(t\right),u\left(t\right)\right)\right]\leq E.$$

### 4.2. Characterization of the Optimal Policy

To search for the stationary optimal policy, we can further introduce another Lagrange multiplier ν≥0 to eliminate the power constraint, i.e.,

$$\frac{1}{T}E_{\pi }\left[\sum_{t=1}^{T}c_{x}\left(x\left(t\right),q\left(t\right),u\left(t\right)\right)+\nu c_{E}\left(x\left(t\right),q\left(t\right),u\left(t\right)\right)\right]-\nu E.$$

The multiplier ν can be viewed as a power penalty, which will increase the Lagrange function once the average power consumption exceeds the constraint. Then minimizing the above Lagrange function for fixed ν becomes an MDP problem without any constraint.

The following lemma ensures that the optimal stationary policy for the MDP problem has a threshold structure.

**Lemma** **1.** *The optimal stationary policy of the unconstrained MDP problem has the threshold structure, i.e., given state (x,q), there exists a threshold $\tau_{q}$ such that if $x\geq \tau_{q}$, then it is optimal to schedule the sensor; otherwise, idling is the optimal action.*

Proof sketch: The complete proof is provided in Appendix B and Appendix C, which is similar to Theorem 2 in [23]. Despite the complex proof, the intuition is simple. As it is optimal to schedule the sensor in state (x,q), then it is also the optimal action in state $\left(x^{\prime },q\right),\;\forall x^{\prime }>x$ because the AoI is much bigger.

### 4.3. Linear Programming Approximation

Now, we focus on finding the optimal stationary policy. Denote $\xi_{x,q}$ to be the scheduling probability given state (x,q) and our goal is to find $\left\{\xi_{x,q}^{*}\right\}$ that minimizes the objective function. In this part, we will approximate $\left\{\xi_{x,q}^{*}\right\}$ by solving a truncated LP.

According to Lemma 1, for the optimal stationary policy, we can set a threshold $X>\max_{q}\tau_{q}$ and then we have $\xi_{x,q}^{*}=1,\;\forall x\geq X$. Next, we focus on searching for policies that possesses this threshold property as other policies are far from optimality.

Let $\mu_{x,q}$ be the steady distribution of state (x,q). Then, define a new variable $y_{x,q}≜\mu_{x,q}\xi_{x,q}$. The following theorem converts the CMDP problem into an infinite LP problem.

**Theorem** **2.** *The single-sensor decoupled problem is equal to the following infinite LP problem.*

(13a) $$\left\{\mu_{x,q}^{*},y_{x,q}^{*}\right\}=\arg\min_{\mu_{x,q},y_{x,q}}\sum_{q=1}^{Q}\sum_{x=1}^{\infty }\left(f\left(x\right)\mu_{x,q}+\lambda y_{x,q}\right),$$

(13b) $$\begin{array}{cc} s.t.\; & \sum_{q=1}^{Q}\sum_{x=1}^{\infty }\mu_{x,q}=1, \end{array}$$

(13c) $$\begin{array}{cc} \; & \mu_{1,q^{\prime }}=\sum_{q=1}^{Q}\sum_{x=1}^{\infty }\left(1-\varepsilon_{q}\right)p_{qq^{\prime }}y_{x,q}, \end{array}$$

(13d) $$\begin{array}{cc} \; & \mu_{x,q^{\prime }}=\sum_{q=1}^{Q}p_{qq^{\prime }}\left[\mu_{x-1,q}-\left(1-\varepsilon_{q}\right)y_{x-1,q}\right],\;\forall x\geq 2, \end{array}$$

(13e) $$\begin{array}{cc} \; & \sum_{q=1}^{Q}\sum_{x=1}^{\infty }y_{x,q}w\left(q\right)\leq E, \end{array}$$

(13f) $$\begin{array}{cc} \; & 0\leq \mu_{x,q}\leq 1,\;0\leq y_{x,q}\leq \mu_{x,q}. \end{array}$$

**Proof.**  Let us consider the average cost of Equation (8a) by using $\mu_{x,q}$ and $y_{x,q}$. Invoking Equation (9), the one step cost of state (x,q) is either f(x)+λ when scheduling or f(x) when idling. Therefore, the time average cost can be computed as follows,

$$\begin{array}{c} \sum_{q=1}^{Q}\sum_{x=1}^{\infty }\mu_{x,q}\left[\xi_{x,q}\left(f\left(x\right)+\lambda \right)+\left(1-\xi_{x,q}\right)f\left(x\right)\right]=\sum_{q=1}^{Q}\sum_{x=1}^{\infty }\left(f\left(x\right)\mu_{x,q}+\lambda y_{x,q}\right), \end{array}$$

which is equivalent to Equation (13a).

Similarly, according to Equation (10), the time average power consumption is

$$\begin{array}{c} \sum_{q=1}^{Q}\sum_{x=1}^{\infty }\mu_{x,q}\left[\xi_{x,q}w\left(q\right)\right]=\sum_{q=1}^{Q}\sum_{x=1}^{\infty }y_{x,q}w\left(q\right), \end{array}$$

which is exactly the LHS of Equation (13e).

Considering the property of steady probability distribution, Equation (13b,f) are verified.

**Figure 1:**
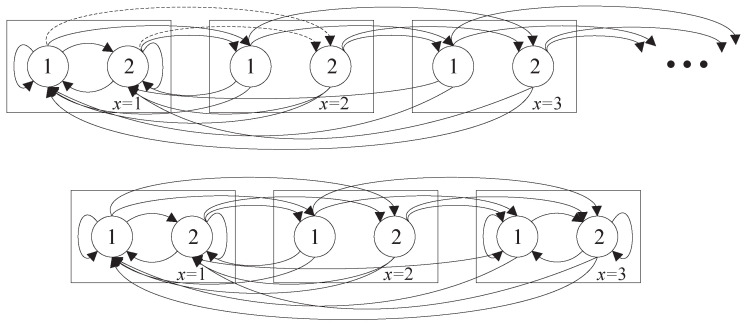
Illustration of the state transition graph for Q=2 channel states without (top) and with (bottom) AoI truncation with AoI threshold X=3. The numbers in circles are channel state index *q*, and the number in rectangles are AoI index *x*.

Finally, notice that the evolution of state (x,q) forms a Markov chain as depicted in Figure 1 (top) for Q=2 as an example. We use $\alpha_{q,q^{\prime }}^{x}=\mathrm{Pr}\left(\left(x+1,q^{\prime }\right)|\left(x,q\right)\right)$ and $\beta_{q,q^{\prime }}^{x}=\mathrm{Pr}\left(\left(1,q^{\prime }\right)|\left(x,q\right)\right)$ to denote the transition probability between the states, which can be computed as follows,

(14) $$\begin{array}{cc} \alpha_{q,q^{\prime }}^{x} & =\left\{\begin{array}{cc} \left(1-\xi_{x,q}\right)p_{qq^{\prime }}+\xi_{x,q}\varepsilon_{q}p_{qq^{\prime }}, & x<X;\\ \varepsilon_{q}p_{qq^{\prime }}, & x\geq X. \end{array}\right. \end{array}$$

(15) $$\begin{array}{cc} \beta_{q,q^{\prime }}^{x} & =\left\{\begin{array}{cc} \xi_{x,q}\left(1-\varepsilon_{q}\right)p_{qq^{\prime }}, & x<X;\\ \left(1-\varepsilon_{q}\right)p_{qq^{\prime }}, & x\geq X. \end{array}\right. \end{array}$$

According to the property of steady distribution, $\mu_{x,q}$ equals to the sum of the steady distribution which can be transferred to $\mu_{x,q}$ in the next slot times their transition probability. As depicted in Figure 1, $\mu_{2,2}=\mu_{1,1}\alpha_{1,2}^{1}+\mu_{1,2}\alpha_{2,2}^{1}$ (see the dashed lines). Therefore, we can compute $\mu_{x,q}$ as follows,

$$\begin{array}{c} \mu_{x,q^{\prime }}=\left\{\begin{array}{cc} \sum_{q=1}^{Q}\sum_{x^{\prime }=1}^{\infty }\beta_{q,q^{\prime }}^{x^{\prime }}\mu_{x^{\prime },q}, & x=1;\\ \sum_{q=1}^{Q}\alpha_{q,q^{\prime }}^{x-1}\mu_{x-1,q}, & x\geq 2, \end{array}\right. \end{array}$$

which is equivalent to Equation (13c,d).    □

As the steady distribution is infinite, it is difficult to solve the problem exactly. Therefore, we approximate the optimization problem in Theorem 2 into a finite LP problem through truncation:

**Problem** **4** (Linear Programming Approximation)**.**

(16a) $$\left\{{\tilde{\mu }}_{x,q}^{*},{\tilde{y}}_{x,q}^{*}\right\}=\arg\min_{\mu_{x,q},y_{x,q}}\sum_{q=1}^{Q}\sum_{x=1}^{X}\left(f\left(x\right)\mu_{x,q}+\lambda y_{x,q}\right),$$

(16b) $$\begin{array}{cc} s.t.\; & \sum_{q=1}^{Q}\sum_{x=1}^{X}\mu_{x,q}=1, \end{array}$$

(16c) $$\begin{array}{cc} \; & \mu_{1,q^{\prime }}=\sum_{q=1}^{Q}\sum_{x=1}^{X}\left(1-\varepsilon_{q}\right)p_{qq^{\prime }}y_{x,q}, \end{array}$$

(16d) $$\begin{array}{cc} \; & \mu_{x,q^{\prime }}=\sum_{q=1}^{Q}p_{qq^{\prime }}\left[\mu_{x-1,q}-\left(1-\varepsilon_{q}\right)y_{x-1,q}\right],\;\forall 2\leq x\leq X-1, \end{array}$$

(16e) $$\begin{array}{cc} \; & \mu_{X,q^{\prime }}=\sum_{q=1}^{Q}p_{qq^{\prime }}\left[\mu_{X-1,q}+\mu_{X,q}-\left(1-\varepsilon_{q}\right)\left(y_{X-1,q}+y_{X,q}\right)\right], \end{array}$$

(16f) $$\begin{array}{cc} \; & \sum_{q=1}^{Q}\sum_{x=1}^{X}y_{x,q}w\left(q\right)\leq E, \end{array}$$

(16g) $$\begin{array}{cc} \; & 0\leq \mu_{x,q}\leq 1,\;0\leq y_{x,q}\leq \mu_{x,q}, \end{array}$$

(16h) $$\begin{array}{cc} \; & y_{X,q}=\mu_{X,q}. \end{array}$$

After truncation, the optimal value of Equation (16a) is the lower bound of the objective function of the decoupled problem Equation (8a). The detailed proof is provided in Appendix D. The key concept is to set a threshold *X* and convert the Markov chain into a finite-state one (see Figure 1).

Moreover, the following theorem guarantees the lower bound obtained by the above LP problem is tight when *X* is sufficiently large. Thus, the approximate optimal solution $\left\{{\tilde{\mu }}_{x,q}^{*},{\tilde{y}}_{x,q}^{*}\right\}$ performs close to the exact optimal solution $\left\{\mu_{x,q}^{*},y_{x,q}^{*}\right\}$. Before displaying the theorem, first denote $\pi_{X}^{*}$ and $\pi^{*}$ to be the scheduling policy according to the approximate optimal solution $\left\{{\tilde{\mu }}_{x,q}^{*},{\tilde{y}}_{x,q}^{*}\right\}$ by setting threshold *X* and optimal one $\left\{\mu_{x,q}^{*},y_{x,q}^{*}\right\}$, respectively. Define $J_{\infty }\left(\pi \right)$ to be the age and scheduling penalty of the primal problem under policy π and $J_{X}\left(\pi \right)$ is the approximate penalty when we set the age penalty f(x)=f(X),∀x≥X. Then, according to Equations (13a) and (16a), we have

$$\begin{array}{c} J_{\infty }\left(\pi^{*}\right)=\sum_{q=1}^{Q}\sum_{x=1}^{\infty }\left(f\left(x\right)\mu_{x,q}^{*}+\lambda y_{x,q}^{*}\right),\;J_{X}\left(\pi_{X}^{*}\right)=\sum_{q=1}^{Q}\sum_{x=1}^{X}\left(f\left(x\right){\tilde{\mu }}_{x,q}^{*}+\lambda {\tilde{y}}_{x,q}^{*}\right). \end{array}$$

**Theorem** **3.** *Assume f(x) satisfies the following property: $\exists \;\epsilon \in [\sqrt{\varepsilon_{\max}},1)$ and constant k>0 such that $f\left(X\right)=k\epsilon^{-X}$ and $f\left(x\right)\leq k\epsilon^{-x},\;\forall x\geq X$, where $\varepsilon_{\max}=\max_{q}\varepsilon_{q}$. Then, we have the following property,*

(17) $$J_{\infty }\left(\pi^{*}\right)-J_{X}\left(\pi_{X}^{*}\right)\leq \frac{k\varepsilon_{\max}^{\frac{X+1}{2}-\tau_{\max}}}{1-\varepsilon_{\max}},$$

*where $\tau_{\max}=\max_{q}\tau_{q}$. As we see the above inequality, the difference between optimal solution of Theorem 2 and Problem 4 converges to 0 as the threshold X becomes sufficiently large.*

The entire proof is provided in Appendix E.

After solving the above LP problem, we can obtain the approximate optimal scheduling probability $\left\{{\tilde{\xi }}_{x,q}^{*}\right\}$ by setting a sufficiently large *X* and computing $\left\{{\tilde{\mu }}_{x,q}^{*}\right\}$ and $\left\{{\tilde{y}}_{x,q}^{*}\right\}$. Moreover, analogical to the threshold structure described in Lemma 1, $\left\{{\tilde{\xi }}_{x,q}^{*}\right\}$ also has the following property.

**Lemma** **2.** For any state (x,q) of each sensor, the optimal scheduling probability $\left\{{\tilde{\xi }}_{x,q}^{*}\right\}$ is non-decreasing with x, i.e.,

$${\tilde{\xi }}_{x_{1},q}^{*}\leq {\tilde{\xi }}_{x_{2},q}^{*},\;\forall x_{1}\leq x_{2}.$$

The proof technique is similar to Lemma 1, so it is omitted here.

## 5. Multi-Sensor Problem Resolution

By now, through relaxing, decoupling and truncation, we have obtained the approximate solution to the single-sensor decoupled problem for fixed scheduling penalty λ. In this section, we will go back to solve the multi-sensor problem, and propose a truncated policy to meet the hard bandwidth constraint in Equation (6b).

### 5.1. The Relaxed Problem Resolution

First, we should choose the optimal λ such that the relaxed bandwidth constraint can be fully leveraged. Denote $g\left(\lambda \right)=\min_{\pi }L\left(\pi ,\lambda \right)$ to be the Lagrange dual function, where we choose the approximate optimal policy $\pi^{*}\left(\lambda \right)$ by solving LP. Then, the dual function can be computed as follows,

$$g\left(\lambda \right)=\frac{1}{N}\left(\sum_{n=1}^{N}g_{n}\left(\lambda \right)-\lambda M\right),$$

where $g_{n}\left(\lambda \right)=\min_{\pi }L_{n}\left(\pi ,\lambda \right)$ is the decoupled dual function for sensor *n*. According to the LP approximation, $g_{n}\left(\lambda \right)$ can be further written out as follows,

$$\begin{array}{c} g_{n}\left(\lambda \right)=X_{n}\left(\lambda \right)+\lambda U_{n}\left(\lambda \right), \end{array}$$

where $X_{n}\left(\lambda \right)$ is the average age penalty bounded by $\sum_{x=1}^{X}\sum_{q=1}^{Q}f\left(x\right){\tilde{\mu }}_{x,q}^{n,*}$, and $U_{n}\left(\lambda \right)$ is the average scheduling probability, which equals to $\sum_{x=1}^{X}{\tilde{y}}_{x,q}^{n,*}$.

According to the work in [24], the optimal Lagrange multiplier $\lambda^{*}$ satisfies

$$\begin{array}{c} \lambda^{*}=\sup\left\{\lambda |\sum_{n=1}^{N}U_{n}\left(\lambda \right)\leq M\right\}. \end{array}$$

If $U_{n}\left(\lambda^{*}\right)=M$, then the optimal policy is just $\pi (\lambda^{*})$. Otherwise, the optimal policy is a mixture of two policies, denoted by $\pi_{l}$ and $\pi_{u}$, which can be computed by

$$\begin{array}{c} \pi_{l}=\lim_{\lambda \to \lambda^{*-}}\pi \left(\lambda \right),\;\pi_{u}=\lim_{\lambda \to \lambda^{*+}}\pi \left(\lambda \right). \end{array}$$

Then, we apply the sub-gradient ascend method to find the optimal solution, where the sub-gradient can be computed as follows,

(18) $$d_{\lambda }g\left(\lambda \right)=\sum_{n=1}^{N}U_{n}\left(\lambda \right)-M=U\left(\lambda \right)-M,$$

where $U\left(\lambda \right)=\sum_{n=1}^{N}U_{n}\left(\lambda \right)$ is the total scheduling probability.

Choose $\lambda_{0}=0$ as the starting point, and compute the average scheduling probability $U(\lambda_{0})$. If $U(\lambda_{0})<M$, then it does not need to consider the bandwidth constraint, and thus the optimal solution has already been solved. Moreover, this optimal solution can also be viewed as the lower bound of the primal optimization problem, i.e.,

$$\mathrm{LB}=\frac{1}{N}\sum_{n=1}^{N}\sum_{x=1}^{X}\sum_{q=1}^{Q}f\left(x\right){\tilde{\mu }}_{x,q}^{n,*}\left(\lambda_{0}\right).$$

Otherwise, we need to increase the scheduling penalty through iterations. The update operation in iteration *k* can be written out as follows,

$$\lambda_{k+1}=\lambda_{k}+t_{k+1}d_{\lambda }g\left(\lambda_{k}\right),$$

where $t_{k+1}$ is the step size in iteration *k*.

Moreover, the step size is determined as follows,

$$\begin{array}{c} t_{k+1}=\left\{\begin{array}{cc} \gamma t_{k}, & d_{\lambda }g\left(\lambda_{k}\right)d_{\lambda }g\left(\lambda_{k-1}\right)<0;\\ t_{k}, & \mathrm{otherwise}, \end{array}\right. \end{array}$$

where γ∈(0,1) is a constant.

The determination of the step size above guarantees the algorithm converges from both sides. Therefore, after running the whole algorithm, we can obtain two different scheduling probabilities $M_{l}$ and $M_{u}$:

(19) $$\begin{array}{cc} M_{l} & =\max_{k}U\left(\lambda_{k}\right),\;s.t.\;d_{\lambda }g\left(\lambda_{k}\right)\leq 0; \end{array}$$

(20) $$\begin{array}{cc} M_{u} & =\min_{k}U\left(\lambda_{k}\right),\;s.t.\;d_{\lambda }g\left(\lambda_{k}\right)\geq 0. \end{array}$$

Their corresponding optimal polices are denoted as $\left\{{\tilde{\mu }}_{x,q}^{n,l},{\tilde{y}}_{x,q}^{n,l}\right\}$ and $\left\{{\tilde{\mu }}_{x,q}^{n,u},{\tilde{y}}_{x,q}^{n,u}\right\}$. Then, the optimal stationary policy can be obtained by mixing these two policies:

$$\left\{{\tilde{\mu }}_{x,q}^{n,*},{\tilde{y}}_{x,q}^{n,*}\right\}=\theta \left\{{\tilde{\mu }}_{x,q}^{n,u},{\tilde{y}}_{x,q}^{n,u}\right\}+\left(1-\theta \right)\left\{{\tilde{\mu }}_{x,q}^{n,l},{\tilde{y}}_{x,q}^{n,l}\right\},$$

where the mixed coefficient can be computed as follows,

$$\theta =\frac{M-M_{l}}{M_{u}-M_{l}}.$$

Now, we have obtained the optimal stationary policy of the relaxed scheduling problem. The algorithm flow chart is listed in Algorithm 1. Once we obtain $\left\{{\tilde{\mu }}_{x,q}^{n,*},{\tilde{y}}_{x,q}^{n,*}\right\}$, the optimal scheduling probability $\left\{{\tilde{\xi }}_{x,q}^{n,*}\right\}$ can be computed as follows,

$$\begin{array}{c} {\tilde{\xi }}_{x,q}^{n,*}=\left\{\begin{array}{cc} 1, & \mathrm{if}\;x>X\;\mathrm{or}\;{\tilde{\mu }}_{x,q}^{n,*}=0\;\mathrm{or}\;{\tilde{\xi }}_{x-1,q}^{n,*}=1;\\ \frac{{\tilde{y}}_{x,q}^{n,*}}{{\tilde{\mu }}_{x,q}^{n,*}}, & \mathrm{otherwise}. \end{array}\right. \end{array}$$

**Algorithm 1** Construction of the optimal stationary policy
 **Initialization:**
$\lambda_{-1}=\lambda_{0}=0$, ϵ, $t_{0}$, γ, $M_{u}$ and $M_{l}$

 **for** each n∈[1,N]
**do**

  compute $\left\{{\tilde{\mu }}_{x,q}^{n,*}\left(\lambda_{0}\right),{\tilde{y}}_{x,q}^{n,*}\left(\lambda_{0}\right)\right\}$ and $U_{n}\left(\lambda_{0}\right)$

 **end for**

 **if**
$d_{\lambda }g\left(\lambda_{0}\right)\leq 0$
**then**

  $\left\{{\tilde{\mu }}_{x,q}^{n,*},{\tilde{y}}_{x,q}^{n,*}\right\}=\left\{{\tilde{\mu }}_{x,q}^{n,*}\left(\lambda_{0}\right),{\tilde{y}}_{x,q}^{n,*}\left(\lambda_{0}\right)\right\}$

 **else**

  k=0

  **while**
$|\lambda_{k}-\lambda_{k-1}|>\epsilon$ or $d_{\lambda }g\left(\lambda_{k}\right)>0$
**do**

   **if**
$d_{\lambda }g\left(\lambda_{k}\right)d_{\lambda }g\left(\lambda_{k-1}\right)<0$
**then**

    $t_{k+1}=\gamma t_{k}$

   **else**

    $t_{k+1}=t_{k}$

   **end if**

   $\lambda_{k+1}=\lambda_{k}+t_{k+1}d_{\lambda }g\left(\lambda_{k}\right)$

   **for** each n∈[1,N]
**do**

    compute $\left\{{\tilde{\mu }}_{x,q}^{n,*}\left(\lambda_{k+1}\right),{\tilde{y}}_{x,q}^{n,*}\left(\lambda_{k+1}\right)\right\}$ and $U_{n}\left(\lambda_{k+1}\right)$

   **end for**

   **if**
$d_{\lambda }g\left(\lambda_{k+1}\right)\geq 0$ and $U\left(\lambda_{k+1}\right)\leq M_{u}$
**then**

    $\left\{{\tilde{\mu }}_{x,q}^{n,u},{\tilde{y}}_{x,q}^{n,u}\right\}=\left\{{\tilde{\mu }}_{x,q}^{n,*}\left(\lambda_{k+1}\right),{\tilde{y}}_{x,q}^{n,*}\left(\lambda_{k+1}\right)\right\}$

    $M_{u}=U\left(\lambda_{k+1}\right)$

   **end if**

   **if**
$d_{\lambda }g\left(\lambda_{k+1}\right)\leq 0$ and $U\left(\lambda_{k+1}\right)\geq M_{l}$
**then**

    $\left\{{\tilde{\mu }}_{x,q}^{n,l},{\tilde{y}}_{x,q}^{n,l}\right\}=\left\{{\tilde{\mu }}_{x,q}^{n,*}\left(\lambda_{k+1}\right),{\tilde{y}}_{x,q}^{n,*}\left(\lambda_{k+1}\right)\right\}$

    $M_{l}=U\left(\lambda_{k+1}\right)$

   **end if**

   k=k+1

  **end while**

  $\theta =\frac{M-M_{l}}{M_{u}-M_{l}}$

  $\left\{{\tilde{\mu }}_{x,q}^{n,*},{\tilde{y}}_{x,q}^{n,*}\right\}=\theta \left\{{\tilde{\mu }}_{x,q}^{n,u},{\tilde{y}}_{x,q}^{n,u}\right\}+\left(1-\theta \right)\left\{{\tilde{\mu }}_{x,q}^{n,l},{\tilde{y}}_{x,q}^{n,l}\right\}$

 **end if**


### 5.2. Truncation for the Hard Bandwidth Constraint

Finally, a bandwidth-truncated policy ${\hat{\pi }}_{X}$ is derived from the optimal stationary policy $\pi_{X}^{*}$ to satisfy the hard bandwidth constraint in Equation (6b). Before introducing the truncated policy, first denote S(t) to be the set of sensors to be scheduled in slot *t*, and |S(t)| is the number of sensors to be scheduled in slot *t*. Then, the construction of ${\hat{\pi }}_{X}$ is carried out as follows.

- In slot *t*, compute the scheduling set S(t) according to the optimal stationary policy $\pi_{X}^{*}$.
- If |S(t)|≤M, then ${\hat{\pi }}_{X}$ schedules all these sensors as $\pi_{X}^{*}$ does.
- If |S(t)|>M, the hard bandwidth constraint is never satisfied. Therefore, ${\hat{\pi }}_{X}$ randomly chooses *M* out of |S(t)| sensors to be scheduled in the current slot.

The following theorem guarantees the asymptotic performance of the truncated policy ${\hat{\pi }}_{X}$ compared with $\pi_{X}^{*}$ on certain conditions.

**Theorem** **4.** Suppose the age penalty function is concave, and let $\kappa =\frac{M}{N}$ be a constant. If all the sensors and their channels are identical, i.e., the power constraint and the channel transition matrix are the same, then the truncated policy ${\hat{\pi }}_{X}$ and the optimal randomized policy $\pi_{X}^{*}$ have the following property,

$$\lim_{N\to \infty }J\left({\hat{\pi }}_{X}\right)-J\left(\pi_{X}^{*}\right)=0.$$

The whole proof is provided in Appendix F.

## 6. Simulation Results

In this section, we provide simulation results to verify the performance of the proposed policy. First, we study the average age penalty performance with different types of sensors with different bandwidth constraint and AoI truncation threshold *X*. Next, we study the detailed scheduling decision of each sensor. The average performance is obtained by simulating $10^{5}$ consecutive slots.

### 6.1. Average Age Penalty Performance

In this part, we demonstrate the average performance of our proposed policy. We consider 4-state channel system, i.e., Q=4. The age penalty function is chosen as f(x)=ln(x) unless otherwise specified. The transition matrix $P_{n}$ for each sensor is the same:

(21) $$P_{n}=\left(\begin{array}{cccc} 0.4 & 0.3 & 0.2 & 0.1\\ 0.25 & 0.3 & 0.25 & 0.2\\ 0.2 & 0.25 & 0.3 & 0.25\\ 0.1 & 0.2 & 0.3 & 0.4 \end{array}\right).$$

Denote $\left\{\eta_{q}\right\}$ to be the steady distribution of the channel state. We consider that for each channel state *q*, the energy consumption w(q)=q. According to [12], the optimal policy to minimize the average AoI performance when all the sensors are identical is a greedy policy, which schedules the *M* sensors with the largest AoI and consumes the average power for each sensor $E_{G}=\frac{M}{N}\sum_{q=1}^{Q}\eta_{q}w\left(q\right)$. Therefore, define $\rho_{n}=\frac{E_{n}}{E_{G}}$ to be the power constraint factor which describes the effects of power consumption constraint $E_{n}$.

**Figure 2:**
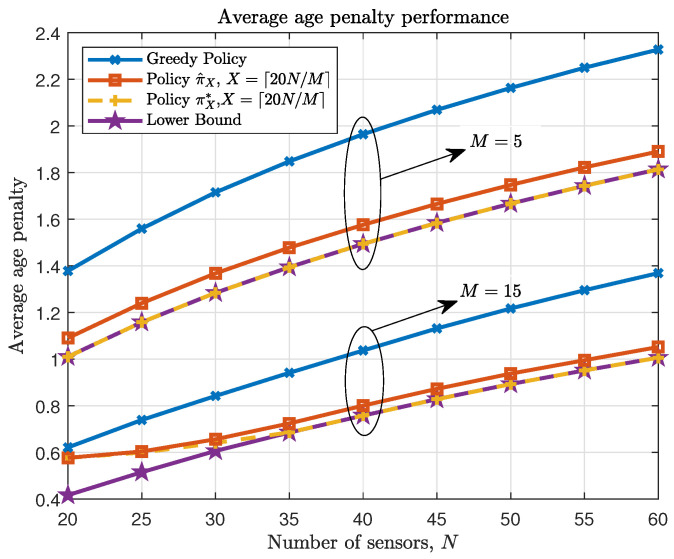
Average age penalty performance as a number of sensors *N*, M={5,15}.

Figure 2 demonstrates the average age penalty performance of the proposed policy ${\hat{\pi }}_{X}$ as a number of sensors *N*, with bandwidth constraint M={5,15}, compared with the lower bound, the relaxed optimal policy $\pi_{X}^{*}$ and the greedy policy. Set the threshold $X=\lceil \frac{20N}{M}\rceil$, where ⌈·⌉ is ceiling function. We assume that the probability of packet loss for each sensor is the same, denoted by ε:

ε=[0.1,0.3,0.2,0.4].

The power constraint factor of sensor *n* is $\rho_{n}=0.2+\frac{1.4(n-1)}{N-1}$.

As seen in Figure 2, the proposed truncated policy performs closely to the relaxed optimal policy and the lower bound, and outperforms the greedy policy especially when *N* is large. According to Figure 2, the age penalty decreases by 18% and 23% from the greedy policy with N=60 sensors when M=5 and M=15, respectively, under proposed policy. Moreover, as the threshold *X* becomes large, the difference between the average performance following policy $\pi_{X}^{*}$ and the lower bound becomes indistinguishable. Therefore, the asymptotic performance described in Theorem 3 can be verified.

**Figure 3:**
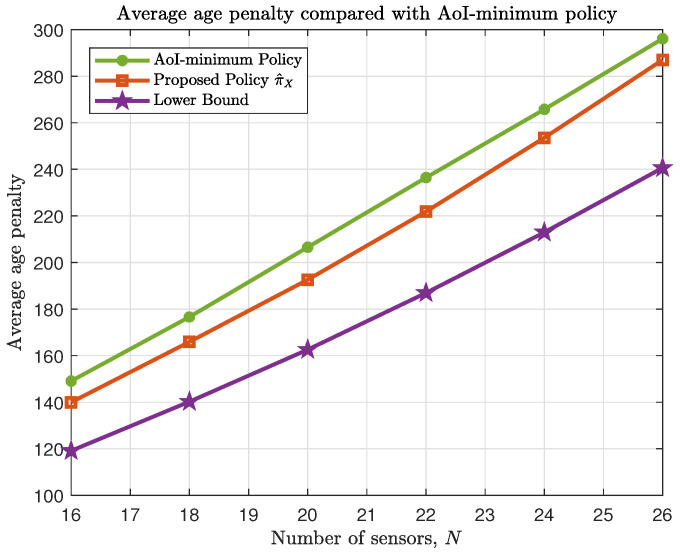
Average age penalty performance as a number of sensors *N* compared with AoI-minimum policy.

Figure 3 compares the average performance of the proposed policy ${\hat{\pi }}_{X}$ with the AoI-minimum policy to verify the improvement of considering different penalty function. The AoI-minimum policy is similar to the one proposed in our previous work [22] with the consideration of packet loss. The bandwidth constraint M=2. The packet loss probability ε=[0.25,0.35,0.65,0.8], and the energy consumption $w\left(q\right)=2^{q}$. Here the age penalty function is chosen as $f\left(x\right)=x^{2}$. From Figure 3, we can see that the AoI-minimum policy cannot guarantee a good age penalty performance. Thus, it is necessary to consider different demand for data freshness to achieve better performance.

**Figure 4:**
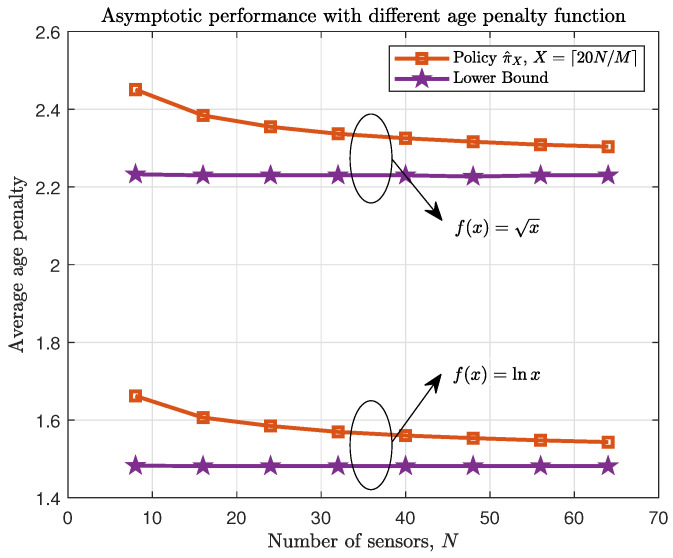
Asymptotic average age penalty performance with different age penalty function $f\left(x\right)=\left\{\ln x,\sqrt{x}\right\}$. $\kappa =\frac{1}{8}$.

**Figure 5:**
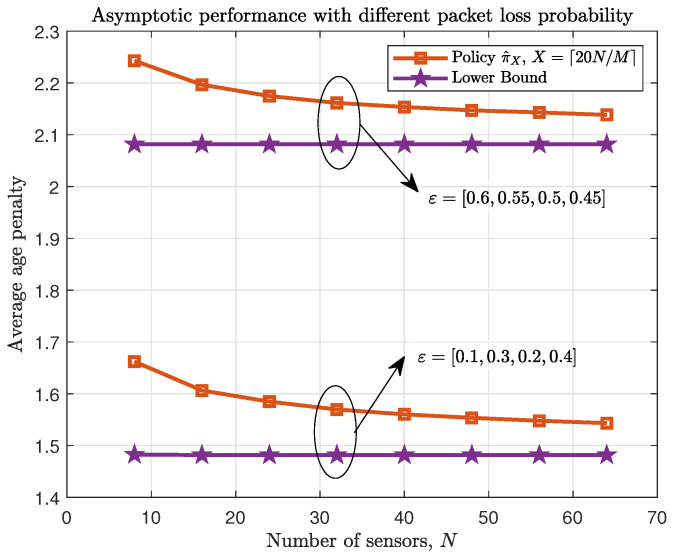
Asymptotic average age penalty performance with different packet loss probability. ε={[0.1,0.3,0.2,0.4],[0.6,0.55,0.5,0.45]}. $\kappa =\frac{1}{8}$.

Figure 4 and Figure 5 verify the asymptotic performance of the proposed policy with different age penalty function and different packet loss probability respectively when M/N is a constant in symmetric networks. From both figures, it can be seen that the difference of average age penalty under proposed policy and the lower bound becomes small as *N* increases. Thus, the asymptotic performance described in Theorem 4 can be verified.

### 6.2. Sensor Level Analysis and Threshold Structure

Next, we analyze the scheduling decision of each sensor and their corresponding age penalty to provide some insights of optimal scheduling policies. We consider a system with N=8 and M=2. The transition matrix of channel state is the same as Equation (21), and power consumption w(q)=q. We set the threshold X=80 to compute the proposed policy.

**Figure 6:**
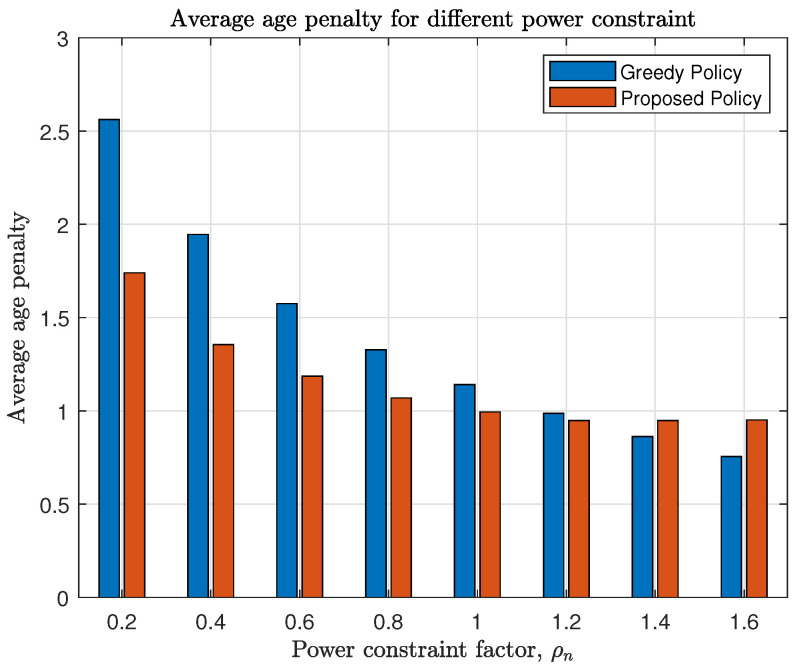
Each sensor’s average penalty with different power constraint factor $\rho_{n}=0.2n$.

First we consider the system with Q=4 and age penalty function f(x)=lnx. Figure 6 analyzes how the power constraint influences age penalty of each sensor. The power constraint of sensor *n* is $\rho_{n}=0.2n$. From Figure 6, we can see that the proposed policy outperforms the greedy policy when the required power consumption is scarce, and performs similarly or a little worse when the factor $\rho_{n}>1$. This implies that our proposed policy chooses a more proper power allocation based on current channel state and AoI than the greedy policy by stimulating sources with scarce power budgets to be scheduled in better channel states.

**Figure 7:**
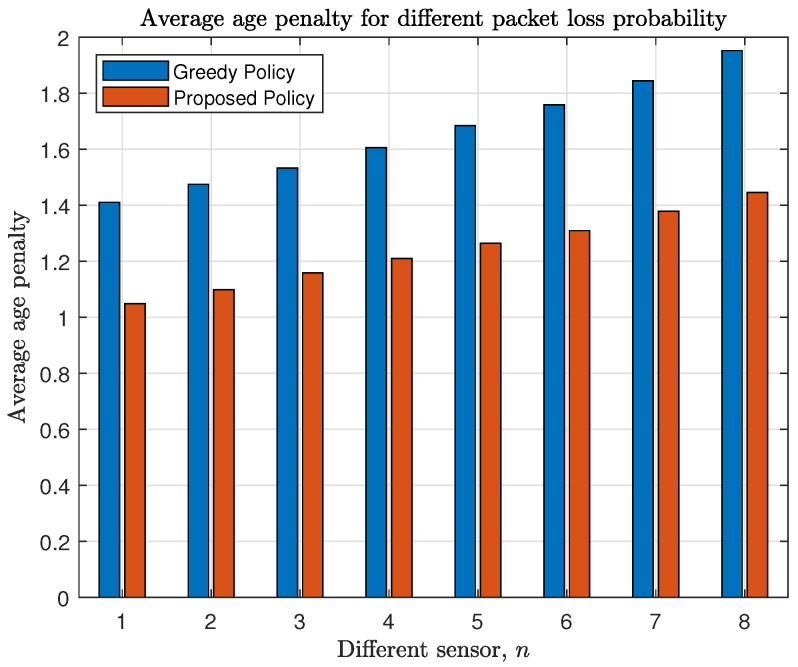
Each sensor’s average penalty with different packet loss probability $\varepsilon_{n,q}=0.05\left(n+q-1\right)$.

As the packet loss influences age penalty as well, Figure 7 considers sensors with different probability of packet loss, which can be written out as the following matrix $\left\{\varepsilon_{n,q}\right\}$:

(22) $$\varepsilon =\left(\begin{array}{cccc} 0.05 & 0.1 & 0.15 & 0.2\\ 0.1 & 0.15 & 0.2 & 0.25\\ 0.15 & 0.2 & 0.25 & 0.3\\ 0.2 & 0.25 & 0.3 & 0.35\\ 0.25 & 0.3 & 0.35 & 0.4\\ 0.3 & 0.35 & 0.4 & 0.45\\ 0.35 & 0.4 & 0.45 & 0.5\\ 0.4 & 0.45 & 0.5 & 0.55 \end{array}\right).$$

We fix the power constraint factor $\rho_{n}=0.6$ for all sensors. Figure 7 shows that the average age penalty increases with the probability of packet loss. Moreover, the proposed policy combats with the packet loss better than the greedy policy as the proposed policy considers $\varepsilon_{n,q}$ when solving the LP problem, but the greedy policy does not.

**Figure 8:**
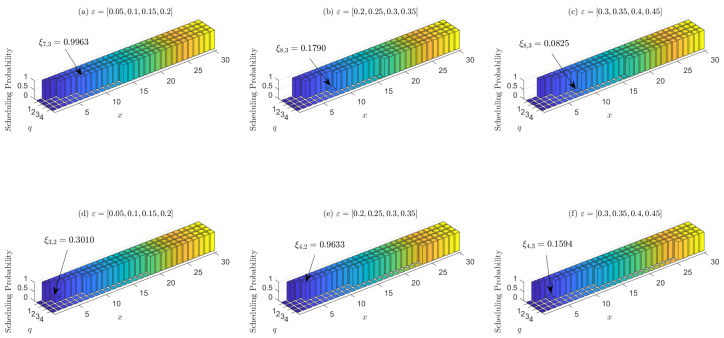
Subfigures (**a**–**c**) demonstrate the scheduling probability of sensors whose packet loss probability is as the title when N=8 and M=2. Subfigures (**d**–**f**) demonstrate their scheduling probability in single-sensor system respectively. All the power constraint is $\rho_{n}=0.6$.

Next, we verify the threshold structure of the optimal scheduling policy. Figure 8 demonstrates the effect of bandwidth and packet loss on the scheduling threshold. The power constraint factor $\rho_{n}=0.6,\;\forall n$, and the packet loss probability is the same as Equation (22). Subfigures (a–c) demonstrate three of these sensors whose packet loss probability is as the title. For each of the three sensors, subfigures (d–f) consider the single-sensor system without bandwidth constraint and display their scheduling probability, respectively. Moreover, Figure 8 lists some of the thresholds given channel state *q*, e.g., in subfigure (a), the threshold of channel state q=3 is x=7, and the corresponding optimal scheduling probability is $\xi_{7,3}=0.9963$. From Figure 8, first we can see that all the six figures verify the non-decreasing property of the scheduling probability with AoI x(t) described in Lemma 2. Second, subfigures (a–c) demonstrate that the sensor with higher packet loss probability also has higher scheduling threshold. This implies that the sensors with more reliable channel should be given higher priority to scheduling than unreliable ones to minimize the average age penalty, since scheduling the more reliable channel under the same AoI is more likely to reduce the current age penalty. Third, by comparing subfigures (a) and (d), (b) and (e), and (c) and (f), the scheduling threshold varies more significantly for different channel states if there exists no bandwidth constraint. The sensors tend to update more often when the channel state is good, and idle when the channel state is bad. This is because the sensors can choose to update packets more frequently in good channel state to both save energy and increase the success probability of transmission without bandwidth constraint.

**Figure 9:**
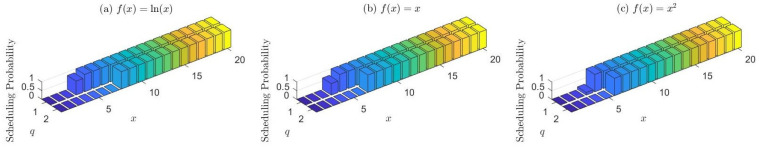
Threshold comparison with different age penalty function.

Finally, we study the effects of age penalty function on threshold structure. Here, we consider a system with Q=2, $\rho_{n}=0.2n$ and three different penalty function, i.e., f(x)=lnx, f(x)=x, and $f\left(x\right)=x^{2}$ in Figure 9. We plot the scheduling decision of the sixth sensor. As is depicted in Figure 9, as the system has a higher restriction on data freshness such as exponential or quadratic function, the difference between thresholds of different states becomes small. In such situations, channel states play a weaker role because waiting for another slot to schedule tends to have unbearable age penalty.

## 7. Conclusions

In this paper, we consider the multi-sensor scheduling problem through an error-prone Markovian channel state. Through relaxing and decoupling, we propose a truncated policy to satisfy both the bandwidth and power constraints to minimize the average age penalty of all sensors in infinite horizon. We prove the asymptotic performance of the truncated policy in symmetric networks when the age penalty function is concave by choosing a sufficiently large threshold *X*. Through theoretical analysis and numerical simulations, we find that the age penalty function, packet loss probability, bandwidth constraint, and power constraint work altogether to influence the optimal scheduling decisions. Those who have more reliable channel state and enough power consumption tend to have higher scheduling priority.

## Appendix A. Proof of Theorem 1

First, we notice that Problem 2 is equivalent to the following optimization problem, where we further introduce variables $\nu_{n}$ to denote the local bandwidth constraint of sensor *n*.

**Problem** **A1** (Equivalent Relaxed Primal Scheduling Problem)**.**

(A1a) $$Age_{R}^{*}=\min_{\pi \in \Pi_{\mathrm{CP}},\left\{\nu_{1},\cdots ,\nu_{N}\right\}}\lim_{T\to \infty }\frac{1}{NT}E_{\pi }\left[\sum_{n=1}^{N}\sum_{t=1}^{T}f\left(x_{n}\left(t\right)\right)\right],$$

(A1b) $$\begin{array}{cc} s.t.\; & \lim_{T\to \infty }\frac{1}{T}E_{\pi }\left[\sum_{t=1}^{T}u_{n}\left(t\right)\right]\leq \nu_{n},\;\forall n, \end{array}$$

(A1c) $$\begin{array}{cc} \; & \sum_{n=1}^{N}\nu_{n}\leq M, \end{array}$$

(A1d) $$\begin{array}{cc} \; & \lim_{T\to \infty }\frac{1}{T}E_{\pi }\left[\sum_{t=1}^{T}u_{n}\left(t\right)w\left(q_{n}\left(t\right)\right)\right]\leq E_{n},\;\forall n, \end{array}$$

(A1e) $$\begin{array}{cc} \; & 0\leq \nu_{n}\leq 1,\;\forall n. \end{array}$$

For each feasible fixed local bandwidth constraint vector $\left\{\nu_{n}\right\}$, we can transfer the above problem into the following one by removing constraint Equations (A1c) and (A1e).

**Problem** **A2** (Relaxed Problem for Fixed $\left\{\nu_{n}\right\}$)**.**

(A2a) $$Age_{R}^{*}\left(\nu_{1},\cdots ,\nu_{n}\right)=\min_{\pi \in \Pi_{\mathrm{CP}}}\lim_{T\to \infty }\frac{1}{NT}E_{\pi }\left[\sum_{n=1}^{N}\sum_{t=1}^{T}f\left(x_{n}\left(t\right)\right)\right],$$

(A2b) $$\begin{array}{cc} s.t.\; & \lim_{T\to \infty }\frac{1}{T}E_{\pi }\left[\sum_{t=1}^{T}u_{n}\left(t\right)\right]\leq \nu_{n},\;\forall n, \end{array}$$

(A2c) $$\begin{array}{cc} \; & \lim_{T\to \infty }\frac{1}{T}E_{\pi }\left[\sum_{t=1}^{T}u_{n}\left(t\right)w\left(q_{n}\left(t\right)\right)\right]\leq E_{n},\;\forall n. \end{array}$$

Then, according to the work in [25], the optimal policy of the Problem 6 given $\left\{\nu_{n}\right\}$ can be decoupled into several local policies. This is somewhat intuitive as the constraints and objective function of Problem 6 are decoupled for each sensor *n*. As for each feasible $\left\{\nu_{n}\right\}$, the optimal scheduling policy can be decoupled, recall that $Age_{R}^{*}=\min_{\nu_{1},\cdots ,\nu_{N}}Age_{R}^{*}\left(\nu_{1},\cdots ,\nu_{N}\right)$, when $\left\{\nu_{n}\right\}$ takes the optimum value $\left\{\nu_{n}^{*}\right\}$, the property also holds.

## Appendix B. Proof of Lemma 1

Before we proceed to the proof, we make two definitions. For any two states s=(x,q) and $s^{\prime }=\left(x^{\prime },q\right)\in S$, define a partial order $\leq_{s}$. We state that $s\leq_{s}s^{\prime }$ if and only if $x\leq x^{\prime }$. Moreover, we also define a partial order $\leq_{a}$ on the action space A. We state that $u\leq_{a}u^{\prime }$ if and only if $u\leq u^{\prime }$.

The monotonicity of the optimal action on the state space is true if the four following conditions hold:

1. If $s\leq_{s}s^{\prime }$, $c\left(s,u\right)\leq c\left(s^{\prime },u\right)$ for any u∈A;
2. If $s\leq_{s}s^{\prime }$, for any u∈A, $\sum_{s_{+}}\mathrm{Pr}\left(s_{+}|s,u\right)V\left(s_{+}\right)\leq \sum_{s_{+}}\mathrm{Pr}\left(s_{+}|s^{\prime },u\right)V\left(s_{+}\right)$,
  where V(s) is any monotone increasing function;
3. If $s\leq_{s}s^{\prime }$ and $u\leq_{a}u^{\prime }$, then $c\left(s,u\right)+c\left(s^{\prime },u^{\prime }\right)\leq c\left(s^{\prime },u\right)+c\left(s,u^{\prime }\right)$;
4. If $s\leq_{s}s^{\prime }$ and $u\leq_{a}u^{\prime }$, then:

$$\begin{array}{cc} \; & \sum_{s_{+}}\mathrm{Pr}\left(s_{+}|s,u\right)V\left(s_{+}\right)+\sum_{s_{+}}\mathrm{Pr}\left(s_{+}|s^{\prime },u^{\prime }\right)V\left(s_{+}\right)\\ \leq & \sum_{s_{+}}\mathrm{Pr}\left(s_{+}|s^{\prime },u\right)V\left(s_{+}\right)+\sum_{s_{+}}\mathrm{Pr}\left(s_{+}|s,u^{\prime }\right)V\left(s_{+}\right); \end{array}$$

where $s,s^{\prime }\in S$, $u,u^{\prime }\in A$. $s^{+}\in S$ is the next state, and c(s,u) denotes the one-step cost given the state *s* and action *u*.

Next, we consider a discounted cost MDP over a finite horizon:

(A3) $$\min_{\pi \in \Pi_{\mathrm{CP}}}E_{\pi }\left[\sum_{k=1}^{T}\beta^{k}c\left(s\left(k\right),u\left(k\right)\right)\right],$$

where β is discounted factor.

And the corresponding Bellman equation is

(A4) $$\begin{array}{cc} \; & V_{T,\beta }\left(s\right)=\min_{u\in A}c\left(s,u\right); \end{array}$$

(A5) $$\begin{array}{cc} \; & V_{k,\beta }\left(s\right)=\min_{u\in A}\left[c\left(s,u\right)+\beta \sum_{s_{+}}\mathrm{Pr}\left(s_{+}|s,u\right)V_{k+1,\beta }\left(s_{+}\right)\right],\;k=1,2,...,T-1. \end{array}$$

If the above four conditions are satisfied and the corresponding Bellman function is monotone increasing, then the one-step cost $c\left(s,u\right)=c_{x}\left(x,q,u\right)+\nu c_{E}\left(x,q,u\right)$ is monotone and sub-modular in *s* and *u*, which shows there exists an optimal monotone policy for any finite-time horizon MDP. Using the vanishing discount approach in Theorem 5.5.4 in [26], the property of monotonicity is propagated to the time-average MDP.

Before verifying the above four conditions, first we introduce the following lemma to ensure $V_{k,\beta }\left(x,q\right)$ is monotone increasing with *x*, whose proof is provided in Appendix C.

**Lemma** **A1.**
*For fixed channel state q and discounted factor β, $V_{k,\beta }\left(x,q\right)$ is monotone increasing with x.*

Therefore, we only need to show the decoupled unconstrained problem satisfies the above four conditions.

Notice that the one-step cost can be computed by

(A6) c(s,u)=f(x)+λu+νw(q)u.

According to the definition of partial order $\leq_{s}$ and $\leq_{a}$, and Equation (A6), we can easily verify condition 1 and 3.

Also, we have:

(A7) $$\begin{array}{c} \sum_{s_{+}}\mathrm{Pr}\left(s_{+}|s,u\right)V\left(s_{+}\right)=\left\{\begin{array}{cc} \sum_{q^{\prime }}p_{qq^{\prime }}V\left(x+1,q^{\prime }\right), & u=0;\\ \sum_{q^{\prime }}\left[p_{qq^{\prime }}\left(1-\varepsilon_{q}\right)V\left(1,q^{\prime }\right)+p_{qq^{\prime }}\varepsilon_{q}V\left(x+1,q^{\prime }\right)\right], & u=1. \end{array}\right. \end{array}$$

According to Equation (A7) and the fact that $V\left(x,q\right)<V\left(x^{\prime },q\right),\;\forall x<x^{\prime }$, it is also feasible to verify that both condition 2 and 4 hold.

## Appendix C. Proof of Lemma A1

In this part, we will prove that $V_{k,\beta }\left(x,q\right)$ in the finite-time horizon MDP is an increasing function with *x*. The key method of the proof is through induction.

Notice that $V_{T,\beta }\left(x,q\right)=\min_{u\in A}c\left(s,u\right)=\min_{u\in A}f\left(x\right)+\lambda u+\nu w\left(q\right)u$ is increasing with *x*. Suppose that $V_{t,\beta }\left(x,q\right)$ is increasing with x,∀t≥k. For $\forall x_{1}<x_{2}$, we have

$$c_{x}\left(x_{1},q,u\right)<c_{x}\left(x_{2},q,u\right),\;c_{E}\left(x_{1},q,u\right)=c_{E}\left(x_{2},q,u\right).$$

Denote $J_{k-1,\beta }\left(x,q,u\right)$ to be the expected discounted cost if take action *u* at state (x,q). Then,

$$\begin{array}{cc} J_{k-1,\beta }\left(x_{1},q,0\right) & =c_{x}\left(x_{1},q,0\right)+\beta \sum_{q^{\prime }}p_{qq^{\prime }}V_{k,\beta }\left(x_{1}+1,q\right)\\ \; & <c_{x}\left(x_{2},q,0\right)+\beta \sum_{q^{\prime }}p_{qq^{\prime }}V_{k,\beta }\left(x_{2}+1,q\right)\\ \; & =J_{k-1,\beta }\left(x_{2},q,0\right). \end{array}$$

Similarly, we can derive that $J_{k-1,\beta }\left(x_{1},q,1\right)<J_{k-1,\beta }\left(x_{2},q,1\right)$. According to the Bellman equation Equations (A4) and (A5), the value function $V_{k-1,\beta }\left(x,q\right)$ can be computed as follows,

$$V_{k-1,\beta }\left(x,q\right)=\min_{u\in A}J_{k-1,\beta }\left(x,q,u\right).$$

From the above, $J_{k-1,\beta }\left(x_{1},q,u\right)<J_{k-1,\beta }\left(x_{2},q,u\right),\;\forall u\in A$. Thus, $V_{k-1,\beta }\left(x_{1},q\right)<V_{k-1,\beta }\left(x_{2},q\right)$. Therefore, through induction, we verify that $V_{k,\beta }\left(x,q\right)$ is increasing with *x*.

## Appendix D. Derivation of Problem 4

First, make the following variable substitution,

(A8) $$\begin{array}{c} \mu_{x,q}^{\prime }=\left\{\begin{array}{cc} \mu_{x,q}, & x<X;\\ \sum_{x^{\prime }=X}^{\infty }\mu_{x^{\prime },q}, & x=X. \end{array}\right. \end{array}$$

Similarly, let

(A9) $$\begin{array}{c} y_{x,q}^{\prime }=\left\{\begin{array}{cc} y_{x,q}, & x<X;\\ \sum_{x^{\prime }=X}^{\infty }y_{x^{\prime },q}, & x=X. \end{array}\right. \end{array}$$

Instead of solving $\mu_{x,q}$ and $y_{x,q}$ in Theorem 2, we solve the new finite variables $\mu_{x,q}^{\prime }$ and $y_{x,q}^{\prime }$. Therefore, the constraints Equation (13b), Equation (13c), Equation (13e), and Equation (13f) become Equation (16b), Equation (16c), Equation (16f), and Equation (16g), respectively. Equation (16d) corresponds to Equation (13d) when x≤X−1. Otherwise, sum all the Equation (13d) from *X* to infinity, i.e.,

$$\begin{array}{cc} \sum_{x=X}^{\infty }\mu_{x,q^{\prime }} & =\sum_{x=X}^{\infty }\sum_{q=1}^{Q}p_{qq^{\prime }}\left[\mu_{x-1,q}-\left(1-\varepsilon_{q}\right)y_{x-1,q}\right]\\ \; & \overset{\left(a\right)}{=}\sum_{q=1}^{Q}p_{qq^{\prime }}\left[\mu_{X-1,q}-\left(1-\varepsilon_{q}\right)y_{X-1,q}\right]\\ \; & \;\;\;+\sum_{x=X}^{\infty }\sum_{q=1}^{Q}p_{qq^{\prime }}\left[\mu_{x,q}-\left(1-\varepsilon_{q}\right)y_{x,q}\right]\\ \; & =\sum_{q=1}^{Q}p_{qq^{\prime }}\left[\mu_{X-1,q}-\left(1-\varepsilon_{q}\right)y_{X-1,q}\right]\\ \; & \;\;\;+\sum_{q=1}^{Q}p_{qq^{\prime }}\left[\sum_{x=X}^{\infty }\mu_{x,q}-\left(1-\varepsilon_{q}\right)\sum_{x=X}^{\infty }y_{x,q}\right], \end{array}$$

where (a) holds because we separate x=X from the sum.

Invoking Equations (A8) and (A9), the above equality is equivalent to

$$\begin{array}{cc} \mu_{X,q^{\prime }}^{\prime } & =\sum_{q=1}^{Q}p_{qq^{\prime }}\left[\mu_{X-1,q}^{\prime }-\left(1-\varepsilon_{q}\right)y_{X-1,q}^{\prime }\right]+\sum_{q=1}^{Q}p_{qq^{\prime }}\left[\mu_{X,q}^{\prime }-\left(1-\varepsilon_{q}\right)y_{X,q}^{\prime }\right]\\ \; & =\sum_{q=1}^{Q}p_{qq^{\prime }}\left[\mu_{X-1,q}^{\prime }+\mu_{X,q}^{\prime }-\left(1-\varepsilon_{q}\right)\left(y_{X-1,q}^{\prime }+y_{X,q}^{\prime }\right)\right], \end{array}$$

which is exactly equal to Equation (16e).

Notice that the optimal action is to schedule when x≥X, i.e., $\xi_{x,q}^{*}=1$. Thus, $y_{x,q}^{*}=\mu_{x,q}^{*},\forall x\geq X$. Therefore, we have

(A10) $$\mu_{X,q}^{\prime }=y_{X,q}^{\prime },$$

which is equal to Equation (16h).

By now, through variable substitution, we have verified the constraints of the finite LP problem are equivalent to the ones of the original optimization problem. Therefore, the optimal solution to the finite LP problem is also feasible to the original problem. Now for the objective function, we have

$$\begin{array}{cc} \; & \sum_{q=1}^{Q}\sum_{x=1}^{\infty }\left(f\left(x\right)\mu_{x,q}+\lambda y_{x,q}\right)\\ = & \sum_{q=1}^{Q}\sum_{x=1}^{X-1}\left(f\left(x\right)\mu_{x,q}+\lambda y_{x,q}\right)+\sum_{q=1}^{Q}\sum_{x=X}^{\infty }\left(f\left(x\right)\mu_{x,q}+\lambda y_{x,q}\right)\\ \geq & \sum_{q=1}^{Q}\sum_{x=1}^{X-1}\left(f\left(x\right)\mu_{x,q}+\lambda y_{x,q}\right)+\sum_{q=1}^{Q}\sum_{x=X}^{\infty }\left(f\left(X\right)\mu_{x,q}+\lambda y_{x,q}\right)\\ = & \sum_{q=1}^{Q}\sum_{x=1}^{X-1}\left(f\left(x\right)\mu_{x,q}^{\prime }+\lambda y_{x,q}^{\prime }\right)+\sum_{q=1}^{Q}\left(f\left(X\right)\mu_{X,q}^{\prime }+\lambda y_{X,q}^{\prime }\right)\\ = & \sum_{q=1}^{Q}\sum_{x=1}^{X}\left(f\left(x\right)\mu_{x,q}^{\prime }+\lambda y_{x,q}^{\prime }\right). \end{array}$$

Therefore, we have verified that the decoupled single-sensor problem is approximate to the LP problem, which has the same constraint but can be the lower bound of the original problem.

## Appendix E. Proof of Theorem 3

Our aim is to bound $J_{\infty }\left(\pi^{*}\right)-J_{X}\left(\pi_{X}^{*}\right)$. Recall that $\pi^{*}$ is the optimal solution to the primal problem. Thus, the difference can be bounded by $J_{\infty }\left(\pi_{X}^{*}\right)-J_{X}\left(\pi_{X}^{*}\right)$.

To further bound the above term, first denote $\rho_{x,q}^{X}$ and $\xi_{x,q}^{X}$ to be the distribution and scheduling probability of state (x,q) under policy $\pi_{X}^{*}$, i.e.,

(A11) $$\begin{array}{cc} \; & \rho_{x,q}^{X}=E_{\pi_{X}^{*}}\left[\lim_{T\to \infty }\frac{1}{T}\sum_{t=1}^{T}I\left((x(t),q(t))=(x,q)\right)\right], \end{array}$$

(A12) $$\begin{array}{cc} \; & \xi_{x,q}^{X}=E_{\pi_{X}^{*}}\left[\lim_{T\to \infty }\frac{1}{T}\sum_{t=1}^{T}I\left(u(t)=1|(x(t),q(t))=(x,q)\right)\right], \end{array}$$

where I(·) is indicating function.

Let $\gamma_{x,q}^{X}=\rho_{x,q}^{X}\xi_{x,q}^{X}$. Recalling the approximation solution to Problem 4, we have

(A13) $$\begin{array}{cc} \; & \rho_{x,q}^{X}={\tilde{\mu }}_{x,q}^{*},\;\forall x<X,\;\sum_{x=X}^{\infty }\rho_{x,q}^{X}={\tilde{\mu }}_{X,q}^{*}; \end{array}$$

(A14) $$\begin{array}{cc} \; & \gamma_{x,q}^{X}={\tilde{y}}_{x,q}^{*},\;\forall x<X,\;\sum_{x=X}^{\infty }\gamma_{x,q}^{X}={\tilde{y}}_{X,q}^{*}. \end{array}$$

Then, we can bound $J_{\infty }\left(\pi_{X}^{*}\right)-J_{X}\left(\pi_{X}^{*}\right)$ as

(A15) $$\begin{array}{cc} \; & J_{\infty }\left(\pi_{X}^{*}\right)-J_{X}\left(\pi_{X}^{*}\right)\\ = & E_{\pi_{X}^{*}}\left[\lim_{T\to \infty }\frac{1}{T}\sum_{t=1}^{T}\left(f\left(x\left(t\right)\right)+\lambda u\left(t\right)\right)\right]-J_{X}\left(\pi_{X}^{*}\right)\\ = & E_{\pi_{X}^{*}}[\lim_{T\to \infty }\frac{1}{T}\sum_{t=1}^{T}\sum_{q=1}^{Q}\sum_{x=1}^{\infty }f\left(x\right)I\left((x(t),q(t))=(x,q)\right)\\ & +\lambda I\left((x(t),q(t))=(x,q),u(t)=1\right)]-J_{X}\left(\pi_{X}^{*}\right)\\ \overset{\left(a\right)}{=} & \sum_{q=1}^{Q}\sum_{x=1}^{\infty }\left(f\left(x\right)\rho_{x,q}^{X}+\lambda \gamma_{x,q}^{X}\right)-J_{X}\left(\pi_{X}^{*}\right)\\ = & \sum_{q=1}^{Q}\sum_{x=1}^{X-1}\left(f\left(x\right)\rho_{x,q}^{X}+\lambda \gamma_{x,q}^{X}\right)+\sum_{q=1}^{Q}\sum_{x=X}^{\infty }\left(f\left(x\right)\rho_{x,q}^{X}+\lambda \gamma_{x,q}^{X}\right)\\ \; & -\sum_{q=1}^{Q}\sum_{x=1}^{X}\left(f\left(x\right){\tilde{\mu }}_{x,q}^{*}+\lambda {\tilde{y}}_{x,q}^{*}\right)\\ \overset{\left(b\right)}{=} & \sum_{x=X}^{\infty }\left(f(x)-f(X)\right)\sum_{q=1}^{Q}\rho_{x,q}^{X}, \end{array}$$

where (a) holds because of Equations (A11) and (A12). (b) holds because of Equations (A13) and (A14).

Next, we upper bound term $\sum_{q=1}^{Q}\rho_{x,q}^{X}$. For simplicity, denote $\rho_{x}^{X}={\left[\rho_{x,1}^{X},\rho_{x,2}^{X},\cdots ,\rho_{x,Q}^{X}\right]}^{T}$. Thus, we have

(A16) $$\rho_{x+1}^{X}=\alpha \rho_{x}^{X},\;\forall x\geq \tau_{\max},$$

where the matrix α can be computed according to Equation (14) and further bounded as follows,

(A17) $$\alpha =\left(\begin{array}{cccc} \varepsilon_{1}p_{1,1} & \varepsilon_{2}p_{2,1} & \cdots & \varepsilon_{Q}p_{Q,1}\\ \varepsilon_{1}p_{1,2} & \varepsilon_{2}p_{2,2} & \cdots & \varepsilon_{Q}p_{Q,2}\\ \cdots & \cdots & \cdots & \cdots \\ \varepsilon_{1}p_{1,Q} & \varepsilon_{2}p_{2,Q} & \cdots & \varepsilon_{Q}p_{Q,Q} \end{array}\right)\leq \varepsilon_{\max}P^{T}.$$

Therefore, $\sum_{q=1}^{Q}\rho_{x,q}^{X}$ can be bounded as follows,

(A18) $$\begin{array}{cc} \sum_{q=1}^{Q}\rho_{x,q}^{X} & =1^{T}\rho_{x}^{X}\\ \; & \overset{\left(a\right)}{=}1^{T}\alpha^{x-\tau_{\max}}\rho_{\tau_{\max}}^{X}\\ \; & \overset{\left(b\right)}{\leq }1^{T}\varepsilon_{\max}^{x-\tau_{\max}}{\left(P^{T}\right)}^{x-\tau_{\max}}\rho_{\tau_{\max}}^{X}\\ \; & \overset{\left(c\right)}{=}\varepsilon_{\max}^{x-\tau_{\max}}, \end{array}$$

where 1 is all-one vector. (a) holds because of Equation (A16). (b) holds due to Equation (A17). (c) holds because 1 is the eigenvector of P.

Plugging Equation (A18) into Equation (A15), we have

$$\begin{array}{cc} J_{\infty }\left(\pi^{*}\right)-J_{X}\left(\pi_{X}^{*}\right)\; & \leq \sum_{x=X}^{\infty }k\left(\epsilon^{-x}-\epsilon^{-X}\right)\varepsilon_{\max}^{x-\tau_{\max}}\\ \; & =\sum_{x=X}^{\infty }k\left(\varepsilon_{\max}^{\frac{x}{2}-\tau_{\max}}-\varepsilon_{\max}^{x-\frac{X}{2}-\tau_{\max}}\right)\\ \; & =\frac{k\varepsilon_{\max}^{\frac{X+1}{2}-\tau_{\max}}}{1-\varepsilon_{\max}}. \end{array}$$

## Appendix F. Proof of Theorem 4

According to Lemma 2, the optimal policy for every decoupled single-sensor problem has the threshold structure. Let $\tau_{q}^{n}$ be the threshold of sensor *n* given channel state *q*. Denote $\Gamma_{n}=\max_{q}\tau_{q}^{n}-\min_{q}\tau_{q}^{n}$ to be the largest difference between different thresholds of sensor *n*, and $\Gamma =\max_{n}\Gamma_{n}$. As all the sensors are identical, Γ does not change with *N*. Moreover, let $\bar{S}\left(t\right)=E_{\pi_{X}^{*}}\left[S\left(t\right)\right]$.

Suppose that the sensor *n* is not scheduled under ${\hat{\pi }}_{X}$ when $u_{n}\left(t\right)=1$. Now consider the probability that it is still not scheduled in the next slot, which results from two reasons, i.e., it jumps into a state which has a higher scheduling threshold or there are still more than *M* sensors to be scheduled. Let *p* be the probability that the channel state jumps into a state having a higher scheduling threshold. Then, the probability of idling in the next slot, denoted by $p_{\mathrm{idle}}$ can be computed by

$$\begin{array}{c} p_{\mathrm{idle}}=p+\left(1-p\right)\frac{N-M}{N}=p\frac{M}{N}+\frac{N-M}{N}. \end{array}$$

Notice that *p* can be upper bounded as

$$\begin{array}{c} p\leq \max_{n,q}\sum_{q^{\prime }=1,q^{\prime }\neq q}^{Q}p_{qq^{\prime }}^{n}=\max_{n,q}\left(1-p_{qq}^{n}\right). \end{array}$$

Therefore, $p_{\mathrm{idle}}$ can be upper bounded by *z*, which can be computed as

$$p_{\mathrm{idle}}\leq z=\frac{M}{N}\max_{n,q}\left(1-p_{qq}^{n}\right)+\frac{N-M}{N}.$$

Therefore, it can be generalized that the probability that it is still not scheduled in the consecutive *k* slots is upper bounded by $z^{{\left(k-\Gamma_{n}\right)}^{+}}$, where ${\left(\cdot \right)}^{+}=\max\left(\cdot ,0\right)$.

Now, we bound the different performance of ${\hat{\pi }}_{X}$ and $\pi_{X}^{*}$ by introducing another policy ${\tilde{\pi }}_{X}$. Under ${\tilde{\pi }}_{X}$, when |S(t)|>M, all these sensors are scheduled like $\pi_{X}^{*}$, but add an extra penalty $a_{x}^{n}\left(t\right)$ for those sensors, which can be computed as

$$\begin{array}{cc} a_{x}^{n}\left(t\right) & =\sum_{k=0}^{\infty }z^{{\left(k-\Gamma_{n}\right)}^{+}}\left(f\left(x_{n}\left(t+k\right)\right)-f\left(k\right)\right)\\ \; & \overset{\left(a\right)}{\leq }\sum_{k=0}^{\infty }z^{{\left(k-\Gamma_{n}\right)}^{+}}\left(f\left(x_{n}\left(t\right)\right)-f\left(0\right)\right)\\ \; & \leq \left(\Gamma_{n}+\frac{1}{1-z}\right)f\left(x_{n}\left(t\right)\right)\\ \; & \leq \left(\Gamma +\frac{1}{1-z}\right)f\left(x_{n}\left(t\right)\right), \end{array}$$

where (a) holds because f(x) is concave.

For simplicity, denote $A=\Gamma +\frac{1}{1-z}$, which is a constant once the channel transition matrix and power constraint are fixed.

As the age penalty function f(x) is concave, the average age penalty cost under ${\tilde{\pi }}_{X}$ does not decrease compared with ${\hat{\pi }}_{X}$. Then, the difference between $J({\hat{\pi }}_{X})$ and $J(\pi_{X}^{*})$ can be bounded as

$$\begin{array}{cc} J\left({\hat{\pi }}_{X}\right)-J\left(\pi_{X}^{*}\right)\leq & J\left({\tilde{\pi }}_{X}\right)-J\left(\pi_{X}^{*}\right)\\ = & \lim_{T\to \infty }E_{\pi_{X}^{*}}\left[\frac{1}{NT}\sum_{n=1}^{N}\sum_{t=1}^{T}a_{x}^{n}\left(t\right)\frac{{\left(|S\left(t\right)|-M\right)}^{+}}{M}\right]\\ \leq & \lim_{T\to \infty }E_{\pi_{X}^{*}}\left[\frac{1}{MNT}\sum_{n=1}^{N}\sum_{t=1}^{T}Af\left(x_{n}\left(t\right)\right)\left||S\left(t\right)|-M\right|\right], \end{array}$$

Notice that when x>X, the optimal action is to schedule. Hence, the probability of $x_{n}\left(t\right)>X$ is upper bounded by ${\left(\max_{n,q}\varepsilon_{n,q}\right)}^{x-X}$. For simplicity, let $\rho =\max_{n,q}\varepsilon_{n,q}$. Then, ∀ϵ>0, there exists $k=\lceil \frac{\ln\epsilon }{\ln\rho }\rceil$ such that the steady distribution $\mu_{x,q}^{n},\;\forall x>X+k$ and *n* can be bounded as

$$\begin{array}{c} \sum_{q=1}^{Q}\mu_{x,q}^{n}\leq \rho^{x-X}=\rho^{k}\rho^{x-(X+k)}\leq \epsilon \rho^{x-(X+k)}. \end{array}$$

Then, we have

(A19) $$\begin{array}{cc} \; & J\left({\hat{\pi }}_{X}\right)-J\left(\pi_{X}^{*}\right)\\ \leq & \lim_{T\to \infty }\frac{1}{MNT}E_{\pi_{X}^{*}}\left[\sum_{n=1}^{N}\sum_{t=1}^{T}I_{x_{n}\left(t\right)\leq X+k}Af\left(x_{n}\left(t\right)\right)\left||S\left(t\right)|-M\right|\right]\\ \; & +\frac{1}{MNT}E_{\pi_{X}^{*}}\left[\sum_{n=1}^{N}\sum_{t=1}^{T}I_{x_{n}\left(t\right)>X+k}Af\left(x_{n}\left(t\right)\right)\left||S\left(t\right)|-M\right|\right]\\ \leq & \lim_{T\to \infty }\frac{Af(X+k)}{M}E_{\pi_{X}^{*}}\left[\frac{1}{T}\sum_{t=1}^{T}\left(\left||S\left(t\right)\right|-\bar{S}\left(t\right)|+|\bar{S}\left(t\right)-M|\right)\right]\\ \; & +\frac{1}{MNT}E_{\pi_{X}^{*}}\left[\sum_{n=1}^{N}\sum_{t=1}^{T}I_{x_{n}\left(t\right)>X+k}ANf\left(x_{n}\left(t\right)\right)\right]\\ = & \lim_{T\to \infty }\frac{Af(X+k)}{\kappa N}E_{\pi_{X}^{*}}\left[\frac{1}{T}\sum_{t=1}^{T}\left(\left||S\left(t\right)\right|-\bar{S}\left(t\right)|+|\bar{S}\left(t\right)-M|\right)\right]\\ \; & +\frac{A}{\kappa }E_{\pi_{X}^{*}}\left[\frac{1}{NT}\sum_{n=1}^{N}\sum_{t=1}^{T}I_{x_{n}\left(t\right)>X+k}f\left(x_{n}\left(t\right)\right)\right]. \end{array}$$

As f(x) is concave, it can be upper bounded by a linear function, i.e., f(x)≤mx,∀x>X+k. Therefore, the second term in the above inequality can be further bounded as

$$\begin{array}{cc} \lim_{T\to \infty }E_{\pi_{X}^{*}}\left[\frac{1}{NT}\sum_{n=1}^{N}\sum_{t=1}^{T}I_{x_{n}\left(t\right)>X+k}f\left(x_{n}\left(t\right)\right)\right]= & \frac{1}{N}\sum_{n=1}^{N}\sum_{x=X+k+1}^{\infty }\sum_{q=1}^{Q}\mu_{x,q}^{n}f\left(x\right)\\ \leq & \sum_{x=X+k+1}^{\infty }m\epsilon \rho^{x-(X+k)}x\\ = & m\epsilon \left[\frac{\rho }{1-\rho }\left(X+k+1\right)+\frac{\rho^{2}}{{\left(1-\rho \right)}^{2}}\right]. \end{array}$$

By choosing $\epsilon =N^{-1}$ and k=O(lnN), the second term in Equation (A19) converges to 0 as *N* becomes large.

For the first term in Equation (A19), according to the work in [27], the expectation of $\left||S\left(t\right)\right|-\bar{S}|$ has the following property,

$$\lim_{T\to \infty }E_{\pi_{X}^{*}}\left[\frac{1}{T}\sum_{t=1}^{T}\left||S\left(t\right)\right|-\bar{S}\left(t\right)|\right]=O\left(\sqrt{N}\right).$$

In addition, as policy $\pi_{X}^{*}$ satisfies the relaxed bandwidth constraint, we have

$$\lim_{T\to \infty }E_{\pi_{X}^{*}}\left[\frac{1}{T}\sum_{t=1}^{T}\left|\bar{S}\left(t\right)-M\right|\right]=0.$$

Therefore, when T→∞, $J\left({\hat{\pi }}_{X}\right)-J\left(\pi_{X}^{*}\right)$ can be upper bounded by

$$\begin{array}{c} O\left(\frac{X+\ln N}{\sqrt{N}}\right)+O\left(\frac{X+1+\ln N}{N}\right). \end{array}$$

As the threshold *X* does not increase with *N*, $J\left({\hat{\pi }}_{X}\right)-J\left(\pi_{X}^{*}\right)$ converges to 0 as *N* becomes infinite. Thus, the asymptotic performance of the truncated policy has been proven.
